# Non-Pharmacological Therapy for Atrial Fibrillation: Managing the Left Atrial Appendage

**DOI:** 10.1155/2012/304626

**Published:** 2012-05-15

**Authors:** Sushil Allen Luis, Damian Roper, Alexander Incani, Karl Poon, Haris Haqqani, Darren L. Walters

**Affiliations:** Department of Cardiology, The Prince Charles Hospital, Rode Road, Chermside, QLD 4032, Australia

## Abstract

The prevalence of atrial fibrillation (AF) is increasing in parallel with an ageing population leading to increased morbidity and mortality. The most feared complication of AF is stroke, with the arrhythmia being responsible for up to 20% of all ischemic strokes. An important contributor to this increased risk of stroke is the left atrial appendage (LAA). A combination of the LAA's unique geometry and atrial fibrillation leads to low blood flow velocity and stasis, which are precursors to thrombus formation. It has been hypothesized for over half a century that excision of the LAA would lead to a reduction in the incidence of stroke. It has only been in the last 20–25 years that the knowledge and technology has been available to safely carry out such a procedure. We now have a number of viable techniques, both surgical and percutaneous, which will be covered in this paper.

## 1. Introduction

Atrial fibrillation (AF) is the most prevalent arrhythmia seen in clinical practice with over 2.2 million people in the United States being affected [1]. Given the association of AF with advancing age, this figure is predicted to increase significantly over the years to come, in line with an aging population. By 40 years of age, the lifetime risk of a man developing AF is 26% and a woman 23% [2]. Although most patients with AF tolerate it well, in a significant proportion of patients the arrhythmia can lead to a substantial reduction in quality of life. The most significant complication, feared by both patient and medical staff, is that of stroke. A stroke in a patient with AF has a poorer prognosis than in a patient without AF [3]. The rhythm had been shown to increase a patient's risk of an ischemic stroke by 4-5 fold [4]. Additionally, AF has been shown to be accountable for up to 20% of all ischemic strokes [5].

Oral anticoagulant therapy, most commonly with warfarin, has been used to reduce the risk of stroke in patients with nonvalvular AF who are at high risk of thromboembolism [6]. Overall, warfarin is underused in these patients mainly due to patient and health practitioner concerns about the increased risk of significant bleeding with aggressive anticoagulation. Clinical data has suggested that only 50–60% of patients who clinically should be prescribed warfarin are actually taking it [7]. Furthermore, clinical trials have demonstrated that a significant proportion of patients who are taking warfarin, are not adequately anticoagulated placing them at an increased risk of stroke. There are now alternative medications to warfarin, which are available. Apixaban is an oral factor Xa inhibitor, which has been shown to be superior to warfarin in preventing stroke or systemic embolism in patients with nonvalvular AF (1.27% per year versus 1.60%; *P* = 0.01 for superiority) [8]. Dabigatran is a direct thrombin inhibitor and is available in two different doses. The Re-ly trial included 18,113 patients with AF and a risk of stroke. Patients were randomized to receive either warfarin, dabigatran 110 mg twice daily or 150 mg twice daily. It showed that the lower 110 mg dose had similar rates of stroke and systemic embolism to warfarin (1.52% per year versus 1.69%; *P* < 0.001 for noninferiority). Furthermore, the higher 150 mg dose was shown to be superior to warfarin (1.11% per year versus 1.69%; *P* < 0.001 for superiority) [9]. These agents tend to have a lower incidence of bleeding with more convenient dosing regimens without the need for intensive monitoring. However, these agents would not benefit those patients who have a contraindication to anticoagulation, which incorporates between 14–44% of patients with AF who are at risk of stroke [10]. Even with adequate anticoagulation, the risk of stroke is not abolished. The annual incidence of stroke in patients therapeutically warfarinised is 2–5% [11]. These issues have led to an increased interest in alternative treatment strategies to help reduce the risk of stroke in these patients. The main focus of this interest is on the left atrial appendage (LAA). It has been shown that more than 90% of thrombi in patients with nonrheumatic, nonvalvular AF are located within this cavity [12]. The LAA forms during the 3rd week of gestation and temporarily acts as the left atrium. An outpouching of the pulmonary veins develops into the true left atrium [13]. The size and morphology of the LAA varies from person to person and has been measured anywhere from 0.77 to 19.27 cm^3^. The LAA is often described as being redundant. A number of studies have shown that it plays an important role in the regulation of the intravascular volume status as well as hemodynamic conditions. Excision of the LAA is thought to lead to a reduction in the incidence of stroke in patients with AF. New techniques have emerged which have enabled this process to be done percutaneously without exposing patients to high-risk invasive surgery. Due to the complexity and variability of the LAA anatomy, multi-modality imaging techniques play an integral role in the workup of these patients and directing percutaneous closure. This is particularly important to ensure correct sizing of a device and reduce the risk of complications such as device embolisation or free-wall perforation.

## 2. Surgical Options

Historically, obliteration of the left atrial appendage was first used in conjunction with mitral valvotomy, prior to the utilization of cardiopulmonary bypass. Madden carried out the first LAA surgical excision in humans in 1949, publishing 2 cases the same year [14]. Initial results were not promising. The first recorded cases had unacceptably high complication rates, and interest in the procedure waned [15]. Interest in surgical treatment of AF reignited when James Cox introduced the Cox-Maze procedure at the Barnes-Jewish Hospital, St. Louis, in 1987. This helps to terminate AF by making multiple deep incisions in both atria in an attempt to block the electrical pathways needed to sustain the arrhythmia. Additionally the Maze procedure usually includes excision or closure of the LAA. A number of improvements have been made since, resulting in the Cox-Maze III procedure, which is still used in clinical practice today [16–19].

The success of the Maze procedure was illustrated in a retrospective analysis by Cox et al. [20] looking at patients who underwent the procedure for AF which had been unsuccessfully treated medically. Three hundred and six patients were included over an 11-year period, of which 58 had either a previous stroke or transient ischemic attack. Two patients were noted to have perioperative strokes (0.7%) and of the 265 patients who were followed up for up to 11 years, only 1 patient was recorded as having had a stroke.

A number of different surgical methods have been used to close the LAA. There are 2 general techniques adopted. Firstly, excision of the LAA can be performed by either removing the LAA and oversewing or a stapled excision. Secondly, exclusion of the LAA can be achieved with sutures or clips on either the endocardial or epicardial surface, isolating the LAA from the left atrium. Additionally, small case series have been reported using a thoracoscopic approach for exclusion of the left atrial appendage without the need for an open sternotomy. The success of each method has been highly scrutinized as clinical evidence has questioned their success.

The Left Atrial Appendage Occlusion Study included 77 patients undergoing CABG [21]. They were randomized to either LAA occlusion or control. The surgical management of the LAA involved either stapled exclusion or suture ligation. The surgery was deemed successful in 72% of the stapled exclusion group compared with 45% in the suture ligation group. The predominant reason for failure in the group that underwent suture ligation appeared to unsatisfactory closure, with persistent Doppler flow within the appendage seen on echocardiography. Furthermore, incomplete closure is likely to be more detrimental than not attempting surgery. A follow-up study looking at patients who have undergone surgical LAA closure showed that 41% of patients with an unsuccessful closure had a thrombus within the LAA on transesophageal echocardiography [22]. The study looked at 137 patients who had either LAA excision (52 patients = 38%) or exclusion (85 patents = 62%), of which 73 were sutured, and 12 were stapled. There was a success rate of only 40% (55/137 patients). Surgical excision was more successful than both suture and staple exclusion (73% versus 23% versus 0%, resp.).

The poor success of certain LAA exclusion techniques had previously been suggested by a study looking at a group of 50 patients who had undergone mitral valve surgery in addition to ligation of the LAA [23]. A transœsophageal echocardiogram was performed at a variable time after procedure, varying from immediately postoperatively to 13 years later. Partial ligation was seen in 36% (18/50 patients) of patients. Of these, 50% (9/18 patients) demonstrated spontaneous echo contrast or thrombus within the LAA, and 22% (4/18 patients) had experienced a thromboembolic event.

The FDA has approved the use of the AtriClip device (Articure, West Chester, OH, USA) for closure of the LAA. It consists of 2 rigid titanium tubes with elastic nitinol springs covered with a knit-braided sheath. The LAA is inserted into the device, which then clips at the base, separating the appendage from the left atrium. The initial trial assessing the safety and efficacy of the device was carried out in Europe [24]. It included 34 patients with AF who were undergoing elective cardiac surgery via a median sternotomy. Patients underwent computed tomography studies to assess the device location as well as looking for evidence of blood flow within the LAA. Although operative mortality was 8.8%, none of the deaths were attributable to the device. All patients had successful LAA closure at 3 months. There were no reports of stroke or transient ischemic attacks during the follow-up period. A further nonrandomized, prospective multicenter trial was performed in the United States called the EXCLUDE trial [25]. Again, the study population was composed of patients who were undergoing primary elective cardiac operations with a median sternotomy. Additionally they had to have either AF or have an increased risk of stroke, as measured by the CHADS2 scoring system (CHADS2 >2) [26]. 71 patients were enrolled across 7 US centers with 1 patient being excluded due to inappropriate LAA anatomy. LAA exclusion was deemed successful in 67 of the remaining 70 patients (96%). By the 3-month followup, 61 patients were able to undergo further imaging by either transesophageal echocardiography or computed tomography. At followup, over 98% of patients had successful exclusion of the LAA. There were no adverse advents attributable to the AtriClip procedure.

Newer surgical techniques are still currently under investigation in an attempt to improve success rates of LAA closure. One such technique involves invaginating the LAA into the left atrium prior to applying a purse-string suture along the base of the LAA [27]. Once this is in place, the LAA is pulled outwards again and the orifice closed with a second running suture. This procedure has only been reported on 8 patients, and followup imaging is still pending; however, the authors remain optimistic about its potential.

Current practice focuses on the excision of the LAA, given its higher success rates. This usually involves a median sternotomy and is usually best used in patients who require an additional cardiac surgery, in light of the associated surgical risks. As techniques improve and procedures become less invasive, this may well change. In light of the paucity of long-term follow-up data following surgical LAA excision and exclusion: further research is required to define long-term outcomes and need for ongoing anticoagulation following these approaches.

## 3. Percutaneous Management

There are a number of devices that are available for closure of the left atrial appendage (see Table 1). Only the WATCHMAN device has been subjected to a randomized controlled trial against a warfarin control group; with remaining devices being assessed by registry data or prospective trials to assess safety and efficacy. Just three of these devices are currently available for clinical use. These include the LARIAT suture delivery system, the WATCHMAN device, and the AMPLATZER Cardiac Plug. The LARIAT suture delivery system is the only device that has, at this time, received both United States Food and Drug Administration (FDA) and European Conformité Européenne (CE) mark regulatory approval. The other two devices carry European CE mark approval for their use. The AMPLATZER Cardiac Plug approval was based on the available registry data. In Australia, the Therapeutic Goods Administration has approved the use of these two devices.

The antiplatelet and anticoagulant regimens used in the clinical trials varied depending on the device (see Table 2).

### 3.1. AMPLATZER Septal Occluder

**Figure 1:**
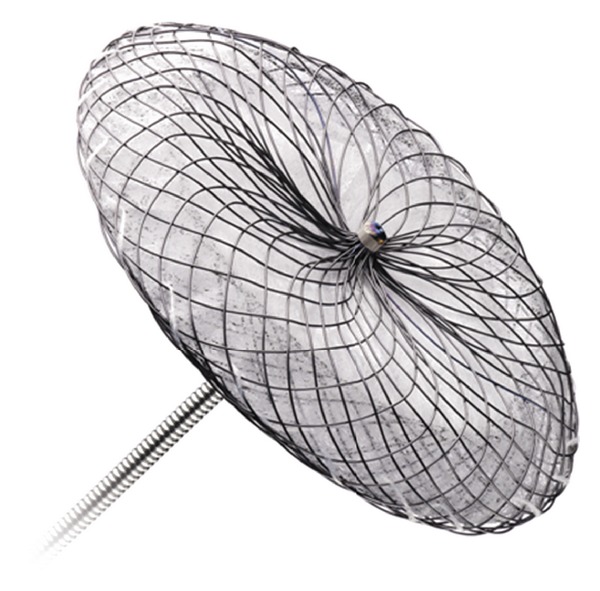
AMPLATZER septal occluder. (AMPLATZER and St. Jude Medical, Inc. Reprinted with permission of St. Jude Medical, © 2011. All rights reserved).

The AMPLATZER Septal Occluder (St. Jude Medical, Plymouth, MN, USA) was initially designed for patent foramen ovale and atrial septal defect closure. A small trial of 16 patients across 4 centres assessed its use in left atrial appendage occlusion [28]. A standard AMPLATZER atrial septal defect occluder with a neck a few millimetres smaller than the neck of the left atrial appendage was selected. The left disk of the occluder was released within the left atrial appendage and right disk released at the left atrial appendage entrance. A single technical failure occurred due to inappropriate device sizing resulting in device embolization and requiring cardiac surgery for device retrieval. Over a short device follow-up period of just 4 months, there were no adverse events attributable to the device implantation or thromboembolic events noted (Figure 1).

No further clinical trials have been conducted using this system for left atrial appendage occlusion. A new device, the AMPLATZER Cardiac Plug, has been designed by the same manufacturer and is currently undergoing clinical trial (see below).

### 3.2. PLAATO

The PLAATO system (eV3, Plymouth, MN, USA) consists of a percutaneously delivered self-expanding nitinol cage covered with an expanded polytetrafluoroethylene and 3 rows of anchors, delivered via a transseptal approach.

Initial assessment in a canine model demonstrated proof of concept with left atrial appendage occlusion being achieved in all cases. Complete healing over the device's membrane surface was demonstrated on both gross and histological examination in 90% by 1 month and in all cases by 3-month follow-up [29].

Subsequent human trials demonstrated a high procedural success rate with successful implantation in 108 of the 111 patients enrolled (97.3%, 95% confidence interval (CI) 92.3% to 99.4%) [30]. Patients were treated with aspirin (300 to 325 mg) indefinitely. North American patients also received 4–6 weeks of Clopidogrel 75 mg daily and subacute endocarditis prophylaxis for the initial six months due to a possible increase in infective endocarditis risk. For European patients, the choice of Clopidogrel and endocarditis prophylaxis was left at the discretion of the treating physician. Unlike subsequent trials, warfarin was not routinely prescribed as part of the trial protocol.

During the initial follow-up period of 90.7 documented implant years covering the initial postimplant period, there were 7 major adverse events in 5 patients [31]. This included 1 episode of cardiac tamponade following transseptal puncture necessitating emergent cardiovascular surgery. This patient subsequently developed a lower limb deep venous thrombosis and died secondary to cerebral haemorrhage thought secondary to anticoagulation [31, 32]. Three other patients underwent in-hospital pericardiocentesis due to a hemopericardium [31]. Other adverse outcomes included 2 strokes occurring at 173 and 215 days postdevice implantation [31]. Their routine 1-and 6-month follow-up transesophageal echocardiograms had demonstrated stable device position with no thrombogenic layer on the device surface; colour flow doppler at six months showed “trace leak” and “absent leak.” Additionally, three TIAs occurred in two patients [31]. There were six deaths in 111 patients, including cardiac or neurological death (*n* = 4), secondary complications after gastrointestinal bleeding (*n* = 1), and an incarcerated hernia (*n* = 1): but none were adjudicated as related to the device or procedure [31].

At 5-year follow-up of the North American cohort of 64 patients, there were 7 (11%) deaths, 5 (8%) major strokes, 3 (5%) minor strokes, 1 (2%) cardiac tamponade requiring surgery, 1 (2%), death from probable cerebral haemorrhage, and 1 (2%) myocardial infarction [32]. Only 1 event (cardiac tamponade) was adjudicated as related to the implant procedure. The annualized stroke/transient ischemic attack (TIA) rate was 3.8%: significantly less than an anticipated stroke/TIA rate of 6.6%/year as estimated by a mean CHADS2 score of 2.6 in the study population [32].

The PLAATO device has been discontinued for commercial reasons.

### 3.3. WATCHMAN

The WATCHMAN device (Atritech, Plymouth, MN, USA) is a percutaneous left atrial appendage closure system delivered via a transseptal approach, and implanted at or immediately distal to the ostium of the left atrial appendage. The device consists of a self-expanding nitinol frame with fixation barbs and a permeable polyester fabric which covers the atrial facing device surface. The WATHMAN device is available in 5 sizes ranging from 21 mm to 33 mm in diameter, to accommodate patient variation in left atrial appendage anatomy and size: with device sizing and placement guided by fluoroscopy and transesophageal echocardiography.

In light of the permeability of the device membrane to blood, warfarin is prescribed with a target International Normalised Ratio (INR) of 2.0 to 3.0 for a period of 45 days postdevice implantation to allow endothelialisation of the device. This is discontinued following this period, if repeat transesophageal echocardiography demonstrates either complete closure of the left atrial appendage or minimal residual peridevice flow, defined as a jet width of <5 mm. Following cessation of warfarin treatment, dual antiplatelet therapy with clopidogrel (75 mg daily) and aspirin (81–325 mg daily) was prescribed until completion of the 6-month follow-up visit, with the use of aspirin alone lifelong thereafter [33].

**Figure 2:**
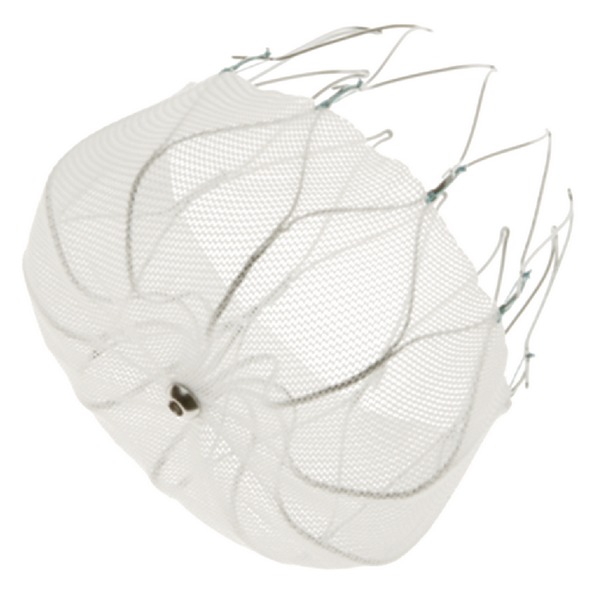
WATCHMAN LAA closure device. (Image courtesy of Atritech, Inc., © 2011).

Evaluation of the WATCHMAN device against a conventionally treated warfarin control population was performed in the PROTECT-AF trial [33], the first trial to randomise device therapy against conventional therapy. The study randomised 707 patients, aged 18 years or older with nonvalvular atrial fibrillation and a CHADS2 risk score of ≥1, in a 2 : 1 intervention to control ratio. An aggregate follow up of 1065 patient-years with mean follow up of 18 months per patient was reported. There was a good procedural success rate, with successful device implantation in 408 (88%) of the 463 randomised to left atrial appendage closure. Following confirmation of left atrial appendage closure on transesophageal echocardiography, warfarin cessation was possible in 86%. Warfarin cessation between 45 days and 6 months was attributable mainly to a reduction in peri-device leak. The WATCHMAN device was determined to be noninferior to conventional warfarin therapy when comparing the primary efficacy endpoint of strokes (ischemic and haemorrhagic), cardiovascular or unexplained death, or systemic embolism. The primary efficacy event rate was 3.0 per 100 patient-years (95% CrI 1.9–4.5) in the intervention group compared with 4.9 per 100 patient-years (95% CrI 2.8–7.1) in control patients receiving warfarin (Figure 2).

However, safety concerns do exist with the placement of the WATCHMAN device: with an excess of adverse outcomes in the WATCHMAN device group compared to warfarinised controls. This is illustrated in the PROTECT-AF trail [33], where the adverse primary safety events occurred at a rate of 7.4 per 100 patient-years (95% CrI 5.5–9.7) in the WATCHMAN group compared with 4.4 per 100 patient-years (95% CrI 2.5–6.7). Most adverse events (55%) were directly related to the procedure occurring on the same day as device implantation. The most frequent adverse outcome was that of a significant pericardial effusion, requiring either percutaneous or surgical drainage, carrying a 4.8% procedural risk. Other adverse procedure related outcomes included major bleeding requiring a transfusion of ≥2 units of packed red cells or surgical intervention (3.5%), procedure-related ischemic stroke (1.1%), device embolization (0.6%), haemorrhagic stroke (0.2%), oesophageal tear (0.2%), and procedure-related arrhythmia (0.2%). However, it should be noted that over the study duration, the incidence of major bleeding (3.5% versus 4.1%) and haemorrhagic stroke (0.2% versus 2.5%) was less in the intervention group compared to the control group. Further published data by Reddy et al. [33] comparing data from the first and second half of the PROTECT-AF trial with the Continued Access Protocol (CAP), suggests that device implantation is associated with a learning phase for the device implanter and that the incidence of adverse safety outcomes have declined with increasing operator experience. The data demonstrates adverse safety outcomes of 10.0%, 5.5%, and 3.7% in the first and second halves of PROTECT AF and CAP, respectively: highlighting a decline in adverse outcome with increasing operator experience [33]. Furthermore, the FDA have mandated a subsequent follow-on randomised controlled trial (PREVAIL), aiming to enrolup to 475 patients with nonvalvular AF with a CHADS2 score of at least 2 AND be eligible for warfarin. This is a higher risk group than used in the PROTECT-AF trial. Patients will be randomised in a 2 : 1 fashion to receive a WATCHMAN device or warfarin only. The primary end-point is a composite of ischaemic/haemorrhagic stroke, systemic embolism and cardiovascular or unexplained death.

Early data from the ASA Plavix (ASAP) registry [34], suggest that in patients with contraindications to warfarin use, the Watchman closure device implantation is possible with the use of 6 months of dual antiplatelet therapy (aspirin and clopidogrel) followed by life-long aspirin thereafter. The registry includes 82 patients at 4 European centers in whom the device was successfully implanted with a median followup is 6 months. In this cohort with limited followup, 2 patients suffered an ischemic stroke. In both cases, no thrombus was demonstrable on the surface of the device or in the left atrium on transesophageal echocardiography. Device-related thrombus was found in 2 other patients on routine follow-up transesophageal echocardiography. Further data including longer-term followup is necessary in order to ascertain the safety and feasibility of this approach in patients with contraindications to warfarinisation.

### 3.4. AMPLATZER Cardiac Plug

The AMPLATZER cardiac plug (St. Jude Medical, Plymouth, MN, USA) was developed based on the AMPLATZER double-disk septal occluders (see above) designed for closure of atrial septal defects and patent foramen ovale. The AMPLATZER cardiac plug system like the other left atrial appendage occluders is designed for implantation through femoral venous access via the transseptal route.

The device consists of a lobe, designed to sit within the left atrial appendage, and an occlusive disc, which fits over the left atrial appendage orifice. The lobe and disc are connected by a central waist, with the lobe containing hooks to ensure device position.

A retrospective review of the preregistry data demonstrated good procedural success with successful device implantation achieved in 132 of the 141 patients (94%) treated [35]. Serious adverse outcomes occurred in 10 (7.0%) patients, including 3 ischemic strokes, 2 device embolization (both recaptured percutaneously), and 5 clinically significant pericardial effusions. Minor complications included 4 clinically nonsignificant pericardial effusions, 2 patients with transient myocardial ischemia, and loss of the implant in the venous system in one patient.

**Figure 3:**
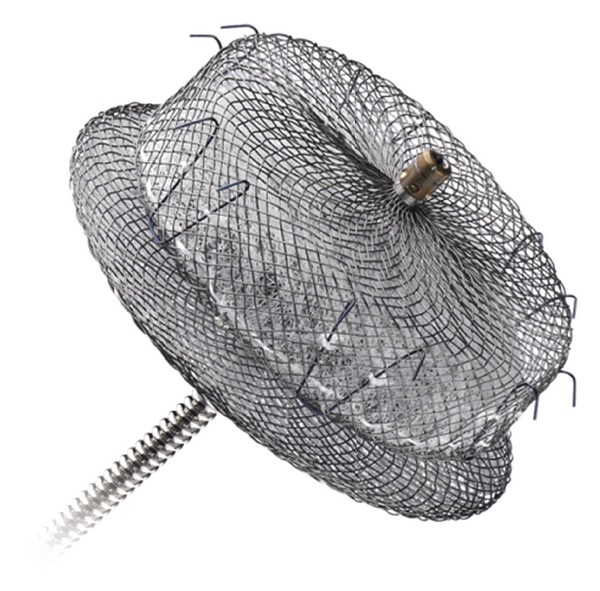
AMPLATZER cardiac plug. (AMPLATZER and St. Jude Medical, Inc. Reprinted with permission of St. Jude Medical, © 2011. All rights reserved).

Interim data from the AMPLATZER Cardiac Plug's European postmarket registry reveals similar procedural success with no device embolizations during the implant procedure. Procedure related adverse events were not significantly changed when compared to the previous data, occurring in 8 out of the 145 registry patients (5.5%) within 7 days after procedure. These consisted of 3 significant pericardial effusions, 3 device embolizations, 1 cardiac perforation, and 1 arteriovenous fistula. Three cases of thrombus on the device and one case of late device embolization were detected during the post 7-day followup (Figure 3).

The AMPLATZER cardiac plug has received European regulatory approval and is available for clinical use in Europe. Additionally, the device is currently undergoing a phase 1 randomised controlled trial compared to conventional warfarin therapy to obtain United States Food and Drug Administration (FDA) approval.

### 3.5. Coherex Wave Crest

The Coherex Wave Crest left atrial appendage occlusion system is a percutaneous transseptally delivered left atrial appendage occluder, consisting of a nitinol frame with retractable coils and anchors to enable optimal device positioning. The device consists of a multicomposite membrane including a porous expanded polytetrafluoroethylene on the left atrial side of the device, nonporous expanded polytetrafluoroethylene barrier layer, and a foam substrate on the left atrial appendage opposing surface to minimise residual leaks.

Initial pilot phase results in 9 patients demonstrated 2 embolic events, prompting device modifications prior to further trials. These modifications included provision of a bidirectional anchor, refined shape, increased radial strength, and improved occluder geometry.

**Figure 4:**
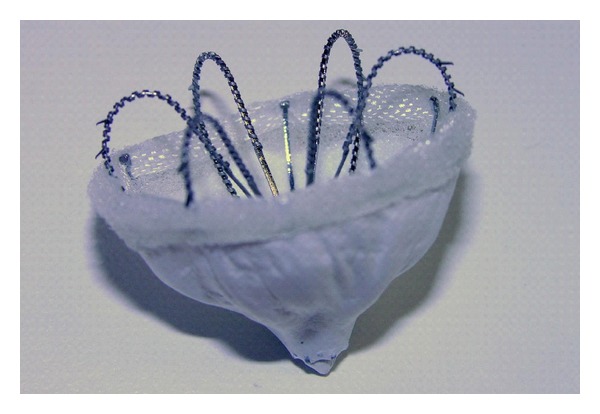
Coherex Wave Crest. (Image courtesy of Coherex Medical).

The Coherex Wave Crest has thus far been implanted in 52 patients. Limited outcome data is currently available with data in a subset of 10 patients treated at a single institution revealing one embolic event with no cases of pericardial effusion or thrombosis [36]. Further clinical trial data regarding device safety and efficacy is currently pending (Figure 4).

### 3.6. LARIAT

The LARIAT procedure (SentreHEART Inc., Palo Alto, CA, USA) for percutaneous left atrial appendage closure requires both *endo*cardial and *epi*cardial access. A preoperative CT is required to exclude large bilobar appendages and other anatomic variants. A magnet-tipped guide wire system is placed at the endocardial surface of the left atrial appendage apex, via femoral venous access and a transseptal puncture. A second magnet-tipped guide wire system is percutaneously introduced into the pericardium overlying the left atrial appendage apex. This requires an anterior approach to percutaneous pericardial access using a 17G Tuohy needle. The magnet-tipped wires, which establish contact easily, align the endocardial and epicardial aspect of the left atrial appendage under fluoroscopy. The LARIAT Suture Delivery Device is guided over the left atrial appendage in an over-the-wire approach to slip a pretied suture loop over the appendage under transesophageal echocardiographic guidance to achieve appendage closure. The LARIAT delivery system can be opened and closed to allow optimal device positioning prior to device deployment [37, 38].

Due to the need for pericardial access, those patients with possible pericardial adhesions are unsuitable for the LARIAT procedure. This includes those with a prior history of cardiac surgery, pericarditis, or chest radiotherapy.

**Figure 5:**
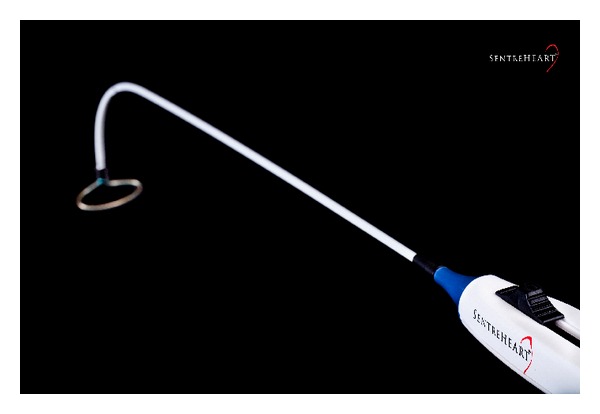
LARIAT (Image courtesy of SentreHEART).

Early human experience demonstrated successful left atrial appendage ligation in 78 of 82 patients undergoing this procedure [39]. The remaining 4 patients suffered from access-related complications including 2 patients with hemopericardium, 1 patient with pericardial adhesions, and 1 patient in whom it was not possible to perform transseptal catheterization. A further 13 were excluded from undergoing this procedure, due to anatomical unsuitability and left atrial appendage thrombus on the preprocedure transesophageal echocardiogram. Of the 70 patients undergoing successful left atrial appendage ligation who had 1 month transesophageal echocardiographic followup, 96% had complete acute closure of the left atrial appendage with the remaining 4% patients having a less than 2 mm jet identified by colour flow Doppler. There were no device-related complications (Figure 5).

No published postprocedural follow-up data is currently available, and further results are currently awaited.

## 4. Conclusions

Surgical methods of LAA closure have been used for more than 20 years, with varying degrees of success. Historically LAA excision has been more successful than exclusion; however, newer surgical techniques are continuously being studied. A number of percutaneous left atrial appendage closure devices are currently undergoing clinical trial. Only three of these device systems are currently available for commercial use. The LARIAT suture delivery system is the only device system with both FDA and European CE mark approval for its use. The remaining two devices (WATCHMAN and AMPLATZER cardiac plug) carry European regulatory approval for this indication. Percutaneous left atrial appendage closure devices are usually associated with high procedural success rates. However, they are also associated with a significant incidence of peri-procedural adverse events; including significant pericardial effusions, major bleeding, procedure-related ischemic stroke, device embolization, and death. Cardiac imaging plays a vital role in assessing anatomical suitability and guiding placement of these devices.

The European Society of Cardiology Guidelines for the management of Atrial Fibrillation suggests that patients with contraindications to chronic oral anticoagulation might be considered as candidates for left atrial appendage occlusion [40]. The 2011 ACCF/AHA/HRS Focused Update on the Management of Patients with Atrial Fibrillation carries no recommendations regarding percutaneous left atrial appendage closure, with the WATCHMAN device noted as currently pending FDA approval [41]. With regards to surgery, the ACC/AHA 2006 Guidelines for the Management of Patients with Valvular Heart Disease recommend patients, undergo amputation of the LAA to when a patient undergoes mitral valve surgery to reduce the incidence of postoperative thromboembolic events [42].

There is a subset of patients who are at a high risk of both cardioembolic stroke and bleeding. It is likely that these patients would be ideal candidates for LAA closure; however, its role still needs to be properly defined.

## Figures and Tables

**Table 1:** Summary of percutaneous devices.

Device	Study	Design	Number of patients	Inclusion criteria	Mean F/U	Results	Comments
LARIAT	Lee et al. [39]	Prospective	82	AF; C/I to warfarin or intolerance to warfarin or pts who have had an embolic event on whilst on warfarin	3 months	96% of patients with successful closure continued to have complete closure at 1 month	(i) Requires both endocardial and epicardial access (ii) Unsuitable in patients with possible pericardial adhesions (e.g., prior history of coronary artery bypass surgery, valvular surgery, pericarditis, and chest radiotherapy) (iii) Clinical trial data pending (iv) FDA and CE mark APPROVED for commercial use

WATCHMAN	Reddy et al. (PROTECT AF) [33]	RCT	707	Permanent or paroxysmal AF; CHADS2 ≥ 1; suitable for warfarin	18 months	Probability of noninferiority of the intervention was more than 99.9%	(i) Efficacy demonstrated in clinical trial (ii) CE mark APPROVED for commercial use

AMPLATZER cardiac plug	Park et al. [35]	Registry	141	Permanent or paroxysmal AF	24 hours after-implantation	Stroke 2.1% Device embolisation 1.4% Pericardial tamponade 3.5%	(i) Clinical trial data pending (ii) CE mark APPROVED for commercial use

AMPLATZER septal occluder	Meier et al. [28]	Prospective	16	Permanent or paroxysmal AF; C/I to warfarin	4 months	TIA/stroke 0% Device embolisation 6.3%	(i) Not a dedicated LAA occluder (ii) Has been superseded by the AMPLATZER Cardiac Plug for LAA occlusion (iii) Not in Commercial use

PLAATO	Ostermayer et al. [30]	Prospective	111	Permanent nonrheumatic AF; patients at risk for stroke; C/I to warfarin	10 months	TIA/stroke 2.2%	(i) No longer available for clinical use
	Block et al. [31]	Prospective	64	Permanent or paroxysmal AF; CHADS2 ≥ 2; C/I to warfarin	5 years	Stroke: 3.8%	

Coherex Wave Crest	Muller (currently recruiting) [36]	Prospective	52- actively recruiting	Permanent or paroxysmal nonvalvular AF; CHADS2 ≥ 1		Data available on 10 cases only, 1 embolic event	(i) Retractable coils and anchors to enable optimal device positioning (ii) Clinical trial data pending (iii) Not in commercial use

C/I: contraindication and AF: atrial fibrillation.

**Table 2:** Summary of antiplatelet/anticoagulation requirements and endocarditis prophylaxis for each percutaneous device.

	Aspirin	Clopidogrel	Warfarin	Endocarditis Prophylaxis
AMPLATZER septal occluder [28]	Few months indefinitely depending on treating centre	None, few months depending on treating centre	None, 6 weeks depending on treating centre	Few months

PLAATO [30]	300–325 mg daily indefinitely	75 mg for 6 months at North American centres and at operator's discretion at European centres	Nil	6 months at North American centres and at operator's discretion at European centres

WATCHMAN [33]	81–325 mg daily indefinitely	75 mg for 6 months	At least 45 days. Discontinued at 45 days if follow up TEE shows <5 mm of peridevice flow	Nil

AMPLATZER cardiac plug [35]	Not specified	Not specified	Not specified	Not specified

Coherex Wave Crest	75–325 mg daily indefinitely	75 mg daily for 90 days if not on warfarin	If previously on warfarin with a history of stroke or TIA, continue warfarin until LAA closure demonstrated on TEE	Nil

LARIAT	Indefinitely	Nil	If previous embolic events whilst on OAC and no contraindication or intolerance, OAC continued regardless of procedural success	Nil

TIA: transient ischaemic attack and OAC: oral anticoagulant.
